# Functions of Differentially Regulated miRNAs in Breast Cancer Progression: Potential Markers for Early Detection and Candidates for Therapy

**DOI:** 10.3390/biomedicines12030691

**Published:** 2024-03-20

**Authors:** Kumar Subramanian, Raghu Sinha

**Affiliations:** Department of Biochemistry and Molecular Biology, Penn State College of Medicine, Hershey, PA 17033, USA

**Keywords:** breast cancer, microRNA, biomarkers

## Abstract

Breast cancer remains a major global health concern, emphasizing the need for reliable biomarkers to enhance early detection and therapeutic interventions. MicroRNAs (miRNAs) are evolutionarily conserved small non-coding RNA (~22 nt in length) molecules, which are aberrantly expressed in cancer and seem to influence tumor behavior and progression. Specific miRNA dysregulation has been associated with breast cancer initiation, proliferation, invasion, and metastasis. Understanding the functional roles of these miRNAs provides valuable insights into the intricate molecular mechanisms underlying breast cancer progression. The diagnostic potential of miRNAs as non-invasive biomarkers for early breast cancer detection is a burgeoning area of research. This review aims to elucidate the functions of differentially regulated miRNAs in breast cancer progression and assess their potential as markers for early detection, stage-specific biomarkers, and therapeutic targets. Furthermore, the ability of specific miRNAs to serve as prognostic indicators and predictors of treatment response highlights their potential clinical utility in guiding personalized therapeutic interventions.

## 1. Introduction

Breast cancer is a disease caused by genetic aberrations and deleterious environmental exposure [1]. Breast cancer is a complex and enigmatic disease caused by a series of alterations in genes that control cell growth and proliferation. Breast cancer is the most common cancer in women and is a heterogeneous disease with diverse molecular subtypes and clinical presentations. Despite advances in treatment, it is the leading cause of cancer mortality in women worldwide. Globally, it accounts for 16 percent of cancer deaths, and has over 20 distinct subtypes that differ genetically, morphologically, and clinically [2,3]. About 90% of these deaths are due to metastases. Currently, breast cancer is the fourth leading cause of cancer-related death, with 2.3 million new cases in 2022 worldwide [4]. The metastatic spread of breast cancer, typically to the bone, lung, liver, and brain, accounts for most cancer-related deaths [5]. There are few treatment strategies for metastatic breast cancer, which is incurable with a median survival rate of 2–3 years [6]. Breast cancer cases are increasing alarmingly, underscoring the importance of treating the disease in many ways. Several trials have been conducted for the successful development of drugs for breast cancers over the past few decades. As a result of inadequate early detection, breast cancer patients have a high mortality rate and recurrence rate. Thus, to improve disease outcomes and prolong patient survival, it is vital to find novel early prognostic and diagnostic biomarkers and effective therapeutic methods [7].

Recent research studies have shed light on the progression of breast cancer, its pathogenesis, and the molecular pathways involved in proliferation. As evidenced by recent research, molecular marker-based targeted therapies using miRNAs may improve the prognosis and diagnosis of a wide range of diseases, including breast cancer [8]. A growing body of evidence suggests that miRNAs play a critical role in tumorigenesis and breast cancer development. These molecules are altered in different tumorigenic processes of breast cancer. miRNAs are small, noncoding RNA molecules that regulate gene expression through interfering with transcription. miRNAs play a significant role in regulating a variety of cellular processes, such as angiogenesis, apoptosis, and the cell cycle. Due to their ability to modulate multiple targets within these pathways, they play a significant role in maintaining cellular homeostasis. It is often observed that dysregulation of miRNAs in these processes contributes to various diseases, including breast cancer, which makes them potential therapeutic targets or diagnostic markers for cancers [9]. It is important to understand the complex network of miRNA-mediated regulation to gain insight into the molecular mechanisms that govern these vital processes within cells.

miRNA was discovered by Ambros and co-workers in *Caenorhabditis elegans* (Nematode), during their genetic study to investigate defects in the temporal control of *C. elegans* development [10]. Recent studies have demonstrated that miRNAs play critical roles in the development of breast cancer, differentiation, proliferation, and other physiological processes [11]. A growing body of evidence suggests that miRNAs might have very important clinical implications.

It is well established that miRNAs are critical regulators of mRNA expression and cell activity, both in normal and abnormal biological processes, including breast cancer. As miRNA dysregulation occurs in various types of cancers including breast cancer and leads to tumor initiation, drug resistance, and metastasis, the therapeutic strategies aimed at modulating miRNA expression levels and identifying their targets are promising strategies [9,11]. miRNAs are secreted by a variety of cells and transported to a variety of bodily fluids in remarkably stable forms (i.e., peripheral blood, saliva, cerebrospinal fluid, ascites, urine, and breast milk) through extracellular vesicles, and interestingly, the levels of miRNAs circulating in cancer patients differ from those of healthy donors [12,13,14,15,16]. Therefore, a quantitative analysis of such potential circulatory miRNAs would be an ideal approach for detecting breast cancer via liquid biopsy in the early stages. This review summarizes the major functions of miRNAs in breast cancer progression and discusses the clinical applications of differentially regulated miRNAs, especially circulating miRNAs in early diagnosis and as targets for therapy.

## 2. miRNA Biogenesis and Maturation

**Figure 1:**
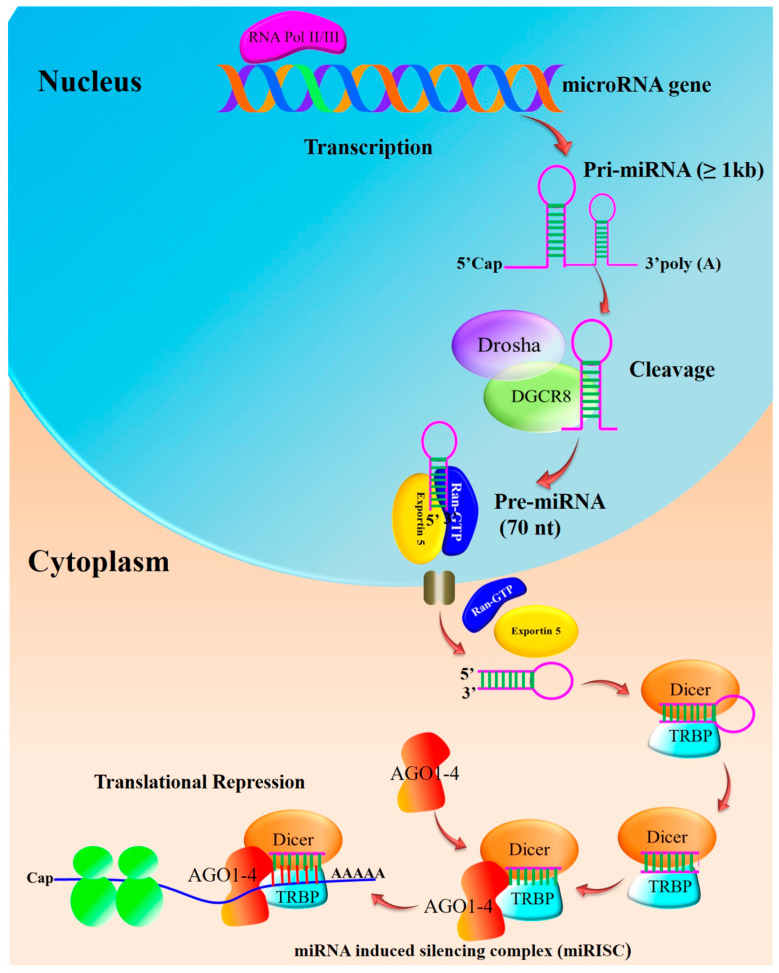
Biosynthesis of miRNA: Pre-miRNAs are further cleaved into an asymmetric duplex using the action of Dicer and accessory proteins. Transactivation-responsive RNA-binding protein (TRBP) and PACT in humans remove the loop sequence by forming a short-lived asymmetric duplex intermediate (miRNA: miRNA), with a duplex of about 22 nucleotides in length. This pre-cursor is cleaved to generate ~21–25-nucleotide mature miRNAs. The mature miRNA is loaded into the miRNA-induced silencing complex (miRISC), which binds to target mRNA, resulting in either the degradation of mRNA or the blockage of translation without mRNA degradation (adapted from [17,18]).

Pri-miRNAs are processed step-by-step in the cytoplasm and nucleus during the biogenesis of miRNAs. In humans, miRNA gene transcription takes place within the nucleus following the cleavage of the ~80 nucleotide stem-loop pre-microRNA precursor performed by the microprocessor complex consisting of Drosha, an RNase III-type nuclease, a double strand RNA-binding protein co-factor, and the DiGeorge syndrome critical region 8 gene (DGCR8) (Figure 1). The complex cleaves the pri-miRNA to generate a shorter hairpin-shaped precursor called precursor miRNA (pre-miRNA). The pri-miRNAs are processed into 60–70 nucleotide hairpin structures (pre-miRNAs) and are exported from the nucleus to the cytoplasm, supported by the nucleocytoplasmic shuttle protein Exportin-5 in a Ran-GTP-dependent manner. The enzyme Dicer further breaks down the pre-miRNA into a double-stranded RNA duplex in the cytoplasm. The mature miRNA is one of the two strands of the duplex that is chosen and loaded into the RNA-induced silencing complex (RISC) [17,18].

By base-pairing with complementary sequences in the 3’ untranslated region (UTR) of the target mRNA, the mature miRNA directs the RISC complex to its target mRNAs. As a result of this interaction, the target gene may be downregulated due to translational repression or mRNA degradation. Multiple steps and molecular players are involved in miRNA biogenesis [17]. Breast cancer has been associated with dysregulation of miRNA biogenesis or its function.

## 3. miRNA Targets and Their Role in Breast Cancer

Discovering the regulatory network regulated by miRNAs requires identification of miRNA-mRNA target interactions. The development of miRNA target prediction algorithms is based on several approaches. Statistical inference based on machine learning and algorithms derived from characteristics of the mRNA sequence and/or based on the miRNA-mRNA interaction are the two major categories. Pairings of miRNA and mRNA seed sequences can be analyzed and evaluated. Instead of making “de novo” predictions based on sequence features, the aim of machine learning is to classify miRNA targets that reference miRNA-mRNA duplexes with known biological significance [19]. miRNA sequences are complementary to 3′-UTR sequences of mRNA targets. For miRNA to bind to target mRNAs, the seed sequence of the miRNA 5′ region is essential. Specific seed region characteristics, as well as those in proximity, have been linked to specific effects on miRNA-induced gene repression [19].

Watson–Crick Pairing between miRNA and mRNA is needed for most target prediction algorithms. miRNA prediction tools include miRNAFinder, miRscan, miRbase, miRTarBase, and SSC profiler [20,21]. Most of them are focused on miRNA conservation characteristics across ecosystems. The prediction of miRNA in a wide range of species from the animal and plant kingdoms have proven successful with this technique.

The miRNAs play an important role in the regulation of gene expression, and the dysregulation of these molecules has been implicated in various stages of breast cancer development. Breast cancer miRNAs are classified based on their expression patterns, functional roles, and clinical implications [22,23]. miRNAs can be classified into two main categories established on their effects on tumorigenesis:

1. Oncogenic miRNAs (OncomiRs): these miRNAs are often upregulated in breast cancer and promote tumorigenesis by inhibiting tumor-suppressor genes or regulatory pathways.
2. Tumor-Suppressive miRNAs: conversely, these miRNAs are downregulated in breast cancer and typically act to inhibit oncogenes or other pro-tumorigenic processes.

To maintain normal cellular function, it is essential to maintain a balance between oncogenic and tumor-suppressive miRNAs as reviewed earlier [24]. In breast cancer, dysregulation of miRNA expression contributes to tumorigenesis (Table 1). Therapeutic potential exists in manipulating miRNA expression. OncomiRs can be inhibited or replaced with miRNAs (for tumor-suppressive miRNAs).

Studying OncomiRs and tumor-suppressive miRNAs in breast cancer is of paramount importance due to several reasons. Interactions between OncomiRs and tumor-suppressive miRNAs provide insight into the molecular mechanisms underlying breast cancer progression. The level of dysregulation of these miRNAs in tumor tissue or bodily fluids correlates with the cancer stage, aggressiveness, and clinical outcome. It is possible to tailor personalized treatment strategies for patients by identifying specific OncomiRs and tumor-suppressive miRNAs associated with breast cancer subtypes or treatment responses.

## 4. Significance of miRNAs in Breast Cancer Development

miRNAs function by binding to the 3′ UTR of target mRNAs, leading to translational repression or mRNA degradation. Breast cancer is characterized by dysregulation of miRNAs and their targets, which contribute to various aspects of cancer initiation, progression, and metastasis. Below is an overview of some key miRNAs and their known roles in the regulation of their targets in breast cancer.

### 4.1. Breast Cancer Initiation and Progression

As a multistep process, cancer initiates and progresses with the gradual transformation of human cells into highly malignant forms through progressive genetic changes [70]. It has been recognized that malignant transformation occurs through successive mutations in specific genes, leading to the activation of oncogenes and the inactivation of tumor-suppressor genes. The activities of these genes may represent the final common pathway using which many carcinogens act [71]. There are three main types of genes that play a role in tumor initiation: proto-oncogenes, tumor-suppressor genes, and genes involved in DNA repair. Changes in the genes, like mutations, amplifications, or deletions, may lead to decoupling of biological mechanism involved in the regulation of normal cell growth and differentiation [72].

Accumulating evidence suggests that cancer stem cells (CSCs) or tumor-initiating cells (TICs) with stem cell-like properties can propagate human tumors with heterogeneous tumor populations in immunodeficient mice, such as human-breast-tumor-initiating cells (BTICs) or breast cancer stem cells (BCSCs) that can regenerate breast tumors in an *in vivo* model. Additionally, BTICs self-renew and asymmetrically divide into differentiated cancer cells, and these are trusted to be accountable for cancer stem-like cells that drive breast tumor formation, recurrence, metastasis, and drug resistance [66,73]. Liu et al. proved that BCSCs are involved in the spontaneous metastases of human breast cancer in mouse breast cancer orthotopic models [74]. Several miRNAs have been implicated in the regulation of CSC properties. The dysregulation of miRNA might contribute to the self-renewal of BCSCs and cancer progression.

The pivotal roles of miRNA-200 are well characterized in breast cancer initiation. For example, miRNA-200 family members are significantly downregulated in CD44^+^, CD24^−low^-lineage human primary BCSCs when compared to non-tumorigenic cancer cells. Moreover, miRNA-200b regulates BCSC growth by directly targeting Suz12, a subunit of a polycomb repressor complex (PRC2) and regulates EMT by repressing the E-cadherin gene. Higher expression of the polycomb protein EZH2, which is important in the stem cell self-renewal capability of embryonic and adult stem cells, has been associated with breast cancer progression [75]. Shimono et al. reported that the expression of the tumor-suppressor miRNA-200c decreased the self-renewal ability of BCSCs *in vitro* and tumor formation ability *in vivo* [76].

Yu et al. showed that let-7 and miRNA-30e were downregulated in BCSCs and SK-3rd mammospheres compared to non-tumorigenic cells or more differentiated cells, respectively. Enforced expression of miRNA-30e inhibited mammosphere formation and tumorigenesis of SK-3rd cells *in vitro* and *in vivo*, respectively, by targeting ITGB3 and UBC9 [51]. The miRNA-128 was found to be downregulated in BCSCs (in CD44^+^, CD24^−/low^, and mammospheres) compared to non-cancerous cells [77]. Furthermore, miRNA-128 is significantly low in chemoresistant BTICs enriched from breast cancer cells (SK-3rd and MCF-7) and primary breast tumors, and that its target *Bmi-1* and *ABCC5* are overexpressed in these cancers. The ectopic expression of miRNA-128 sensitizes BTICs to the proapoptotic and DNA-damaging effects of doxorubicin, showing its therapeutic potential [77]. In another finding, miRNA-30c regulates invasion of breast cancer by targeting the cytoskeleton network genes encoding Twinfilin 1 (TWF1) and Vimentin (VIM), both of which regulate EMT [78].

The miRNA-34c expression level and function were reduced in the BTICs of MCF-7 and SK-3rd cells, and these cell lines were enriched for BTICs [79]. When ectopic expression of miRNA-34c decreased the self-renewal of BTICs, inhibited EMT, and suppressed migration of the tumor cells through inhibition of the target gene *Notch4*, miRNA-495 was significantly upregulated in CD44^+^/CD24^−/low^ BCSCs, reflecting its potential importance in maintaining common BCSC properties, and its ectopic expression promoted colony formation *in vitro* and tumorigenesis in mice. In addition, the overexpression of miRNA-495 is associated with the lowered expression of E-cadherin, which enhances the stem-like phenotype in BCSCs [80]. miRNA-181 family members and miRNA-155 oncogenic miRNAs promote self-renewal, sphere formation, colony formation, or tumor development in breast cancer cells [81].

### 4.2. miRNA in Metastatic Breast Cancer

Metastatic breast cancer is a complex, multistep malignant process through which tumor cells migrate from their primary tumor (site of origin) to colonize distant tissues (e.g., liver, brain, bones, or lungs) and is often responsible for 90% of cancer-related mortality [82,83]. EMT is one of the important processes by which cancer metastasis starts, and EMT induces morphological changes in epithelial cells by which epithelial cells transform into mesenchymal cells. During this process, cancer cells lose their cell–cell communication and become mobile and invasive, spreading into distant organs and tissues [84]. Suppression of E-cadherin expression in epithelial cancer cells is a hallmark of EMT. Various miRNAs have been linked to the control of EMT in cancer, and several genes, including *ZEB* [85], *Twist* [86], *Snail* [87], and *Slug* [88] are known to restrict the expression of E-cadherin.

One of the most important miRNAs that control metastasis is the miRNA-200 family (miRNA-200a/200b/200c/141/429), and this prevents cell migration and invasion by targeting *ZEB* in several cancer types, including breast cancer [89], and miRNA-200/ZEB plays a central role in the EMT/MET processes. In the meantime, inhibition of miRNA-200 reduces the E-cadherin level while supporting VIM expression, thereby increasing cell motility [89]. Many studies also demonstrated that TGF-β-induced EMT might be inhibited only with the ectopic expression of the miRNA-200 family [90]. Xie et al. reported that miRNA-193a-*WT1* interaction plays an important role in breast cancer metastasis and indicates that restoring miRNA-193a expression is a therapeutic strategy in breast cancer [91].

Ma and co-workers discovered that upregulation of miRNA-10b promotes invasion and metastasis by indirectly activating the pro-metastatic gene *RHOC* through repressing *HOXD10* [25]. In addition, miRNA-373 regulates the *CD44* gene, which promotes tumor invasion and metastasis [44]. miRNA-9, a *MYC*/*MYCN*-induced miRNA, has been demonstrated to directly target E-cadherin to promote breast cancer metastasis via activation of β-catenin signaling, through increasing tumor invasiveness and angiogenesis [92].

Recently, several studies have reported that Dicer, an endonuclease that processes miRNAs, is also associated with EMT and metastatic progression. Dicer inhibition promotes metastasis, and restoring its expression suppresses metastasis. Certain miRNAs also target Dicer for controlling metastasis. For example, miRNA-103/107 induced EMT by targeting Dicer expression [93]. In addition, the miRNA-221/222 cluster has been shown to induce EMT in breast cancer cells by targeting Dicer, and estrogen receptor 1 (ESR1) [94]. In breast cancer, miRNA-107 acts as an endogenous suppressor of let-7, and it interacts with mature let-7 to inhibit its function, which plays a major role in determining metastatic progression [95]. According to Huang et al., both *in vitro* and *in vivo* cancer cell migration and invasion were promoted by miRNA-373 and miRNA-520c [44]. Wu et al. reported that in breast cancer cells, miRNA-29a regulates the critical roles of EMT and metastasis by targeting *SUV420H2* [96].

The overexpression of miRNA-17/92 is also involved in metastatic breast cancer [27]. miRNA-454-3p plays an important role in breast cancer’s early metastatic events, promotes the stemness of breast cancer cells, and promotes early distant relapse in both *in vitro* and *in vivo* conditions. The higher expression of miRNA-454-3p was found to be significantly associated with both a poor prognosis and early recurrence in breast cancer through Wnt/β-catenin signaling activation [97]. Higher expression of miRNA-373 was found in breast cancer samples from tumors exhibiting lymph node metastasis [44]. Dobson et al. identified a novel target of miRNA-30c, the nephroblastoma overexpressed gene (*NOV*), which is an inhibitor of the invasiveness of metastatic TNBC (MDA-MB-231) cells [98]. The miRNA-34a plays a key role in the proliferation, invasion, and metastasis of breast cancer cells [53]. Moreover, miRNA-373, miRNA-520c, miRNA-210, and miRNA-29b were also shown to influence the invasion and migration of breast cancer [44]. In addition, oncogenic miRNA-224 expression is significantly upregulated in highly invasive MDA-MB-231 cells and correlated with increased metastasis [99]. Another important oncogenic miRNA, miRNA-155, is frequently overexpressed in invasive breast cancer tissues and is directly targeted to *RhoA* and contributes to breast cancer metastasis. Inhibition of miRNA-155 suppressed TGF-β-induced EMT and tight junction dissolution, along with cell migration and invasion. Further, the ectopic expression of miRNA-155 reduced RhoA protein and disrupted tight junction formation [100]. miRNA-21 is an important oncogene and has a role in breast cancer tumorigenesis. It regulates invasion and tumor metastasis by targeting multiple tumor and metastasis suppressor genes. Also, inhibiting miRNA-21 reduced the invasion and lung metastasis of MDA-MB-231 cells [30]. All these studies demonstrated that some of the miRNAs have a metastatic role in breast cancer cells.

## 5. miRNA Expression in Breast Cancer Subtypes

Breast cancer comprises a diverse pathophysiology, with several molecular subtypes exhibiting varying clinical features, therapeutic responses, and prognoses. The immunohistochemical expression of hormone receptors such as the estrogen receptor (ER), progesterone receptor (PR), and human epidermal growth factor receptor 2 (HER2), is a widely accepted and commonly used classification system for breast cancer [101,102,103]. There are ER^+^/PR^+^, HER^+^, or triple negative (ER^−^, PR^−^, HER2^−^, i.e., does not express these three receptors) clinical definitions for breast tumors. Further, breast cancer can be subtyped including Luminal A (ER^+^ PR^high^ HER2^−^ Ki-67^low^), Luminal B (ER^+^ PR^+^ HER2^+^ or HER2^−^ Ki-67^high^), HER2-enriched (ER^−^ PR^−^ HER2^+^), and triple negative or basal-like (ER^−^ PR^−^ HER2^−^) [104]. Dysregulation of miRNAs has been associated with breast cancer development at various stages. In addition to these markers, miRNAs have emerged as additional biomarkers that contribute to the molecular and functional diversity of breast cancers [22,23]. miRNAs in breast cancer are classified based on their expression patterns, functional roles, and clinical implications. Table 2 lists some miRNA biomarkers associated with breast cancer molecular subtypes. This classification allows us to stratify breast cancers according to their molecular subtype, which guides the diagnosis and treatment decisions and predicts prognosis. As far as the treatment of breast cancer is concerned, two main targeted therapies are commonly used: hormone therapy for hormone receptor-positive tumors [105] and anti-HER2 therapy for HER2^+^ tumors [106]. It is anticipated that future studies will identify miRNA biomarkers and their associations with specific subtypes of cancer. In breast cancer, miRNA profiling can improve molecular subtyping accuracy and guide better personalized treatment approaches.

## 6. miRNAs as Potential Diagnostic Biomarkers for Breast Cancer

In recent years, investigating biomarkers for early detection has rapidly expanded for better diagnoses and prognoses and the prediction of treatment responses in breast cancer. Using significant features, including stability, tissue specificity, ease of detection, and manipulation, will attract the clinician’s attention to achieve personalized breast cancer treatment [127]. miRNA are emerging, attractive, and promising biomarkers owing to their detectability and stability in the bloodstream. Usually, a significant increase in miRNA level is observed in cancer compared to controls; therefore, diagnostic and monitoring applications for miRNAs must be examined. Determining the expression ratios of genes and miRNAs has been a useful technique to improve diagnostic potential. The circulating miRNAs are widely known to be relatively stable, accessible, less invasive, easy to test, promising, and stage-specific biomarkers for the non-invasive diagnosis of breast cancer [128]. miRNAs are exceptionally stable in the bloodstream against the action of endogenous RNases, suggesting that miRNAs may serve as an effective blood-based biomarker for the detection and diagnosis of cancer [129]. miRNA expression has been observed to be higher in tumors than in normal tissues, leading us to hypothesize that global miRNA expression reflects the level of differentiation of cells. Tumor tissues release miRNAs into the bloodstream and other body fluids. miRNA levels may reflect abnormal conditions in breast cancer [130]. Therefore, miRNA status in the body fluids could be a novel, specific, and very sensitive blood-based diagnostic tool for the early detection of breast cancer. Cancer-specific miRNAs are shedding light on the molecular basis of cancer by being able to identify cancer-specific expression of miRNAs in the blood, which plays a vital role in the early-stage diagnosis of breast cancer. Currently, blood-based biomarkers are widely used for the early diagnosis of breast cancer and in response to treatment [130,131].

Using microarrays and other conventional methods, it is possible to detect differences in miRNA expression patterns between normal and cancerous tissue. Studies have shown that the profiles of miRNAs can be correlated with different types and grades of tumors, and that these profiles may be different from those of the normal breast tissue and cells [132]. Furthermore, exosome-derived miRNAs may influence the evolution of tumors by serving as messengers between tumor cells and non-tumor cells like immune and stromal cells. This phenomenon was extensively covered in a recent review on the emerging role of exosomes in breast cancer progression [133].

The detection of circulating miRNA can be a useful method for the non-invasive detection of breast cancer biomarkers. Because circulating miRNAs are very stable in clinical sources such as sputum, plasma, serum, urine, and saliva, they have been extensively used to classify breast cancer subtypes more accurately than circulatory cell-free DNA or RNA [134].

The miRNA-21 gene is an important regulator of breast carcinogenesis and is the most sensitive (87.6%) and specific (87.3%) biomarker for breast cancer diagnoses at early stages compared to other biomarkers like CEA and CA153 [135]. Canatan et al. reported that miRNA-21, miRNA-125, and are reliable candidates for circulating miRNA biomarkers for the detection of breast cancer [136]. On the other hand, the serum miRNA expression profile analysis using highly sensitive microarrays revealed five miRNA signatures (miRNA-1246, miRNA-1307-3p, miRNA-4634, miRNA-6861-5p, and miRNA-6876-5p) that can be used to detect early-stage breast cancer [137]. miRNA-195 and let-7a had significantly higher levels in the circulating blood of the breast cancer cohort than in the healthy controls. However, circulating levels of miRNA-195 and let-7a decreased in cancer patients following curative tumor resection [138]. Similarly, preoperative serum miRNA-20a and miRNA-21 expression levels were significantly higher in patients with breast cancer and benign disease than in healthy women. Serum miRNA-214 levels, on the other hand, could distinguish between benign and malignant tumors and healthy controls. In addition, in postoperative serum samples, miRNA-214 levels significantly decreased as compared to the preoperative sample [139].

Breast cancer patients with lymph node metastasis have high levels of miRNA-10b and miRNA-373 circulating in their blood, and their expression is associated with promoting the migration and invasion of breast cancer cells. Furthermore, such miRNAs may serve as viable biomarkers for detecting lymph node metastases in individuals with breast cancer [140]. Some studies revealed that groups of circulating miRNAs such as miRNA-299-5p, miRNA-411, miRNA-215, and miRNA-452 were differentially expressed in metastatic patients and increased the expression of miRNA-20a, miRNA-214, and miRNA-210 in lymph node-positive patient subgroups [139,141,142].

A few studies revealed significant high expression levels of circulatory miRNA-10b, miRNA-34a, miRNA-155, and miRNA-122 in breast cancer patients, which are associated with primary metastatic breast cancer [143]. Similarly, there were significantly higher expression levels of serum miRNA-21, miRNA-29a, miRNA-130b-5p, miRNA-145, miRNA-151a-5p, miRNA-206, miRNA-222-3p, and miRNA-451 in the breast cancer group than in the control group [144,145]. Cuk et al. also identified dysregulated miRNAs (miRNA-148b, miRNA-376c, miRNA-409-3p, and miRNA-801) in the plasma of 127 sporadic breast cancer patients and 80 healthy controls using RT-qPCR [146]. Other studies revealed some of the candidate biomarkers in plasma, such as miRNA-16, miRNA-21, miRNA-451, miRNA-409-3p, and miRNA-652, in the plasma of breast cancer patients [147]. But interestingly, these miRNAs were downregulated after surgery.

## 7. miRNA-Based Therapeutic Approaches to Breast Cancer Treatment

In the field of medicine, miRNAs have gained increasing interest in recent years due to their potential as therapeutic targets. Despite their promise, miRNA-based therapeutics face significant challenges, including issues related to delivery, off-target effects, and the need for rigorous clinical trials. miRNAs are currently being researched to unlock their full therapeutic potential.

For integrated cancer care, miRNA-based gene therapy provides an appealing anti-tumor approach. miRNA expression imbalance in cancer is related to tumorigenesis, so miRNA-based therapies are based on restoring miRNA function or inhibiting overexpressed miRNAs as reviewed earlier [148]. Recent experiments have shown that the phenotype in cancer cells can be reversed and the gene regulation network and signaling pathways can be restored by correcting miRNA changes with miRNA mimics or antagomiRs [149].

There are two different strategies and challenges to the clinical application of miRNAs. One strategy is designed to inhibit oncogenic miRNAs using miRNA antagonists, such as locked-nucleic acids (LNA), or antagomiRs, and is aimed at gaining function. To inhibit miRNA activity, small-molecule inhibitors specific to certain miRNAs are also being developed. The second strategy, miRNA replacement, includes the reintroduction of tumor-suppressor miRNA mimics to restore the loss of function [149,150,151,152].

### 7.1. miRNA Inhibition Therapy

By repressing oncogenic miRNAs, we can inhibit tumorigenesis, which is a promising cancer treatment strategy. Oncogenic miRNAs are often overexpressed in breast cancer and need to be suppressed to restore the normal expression of their target tumor-suppressor genes. In the recent past, multiple strategies have been developed to inhibit oncogenic miRNAs [153,154].

**Figure 2:**
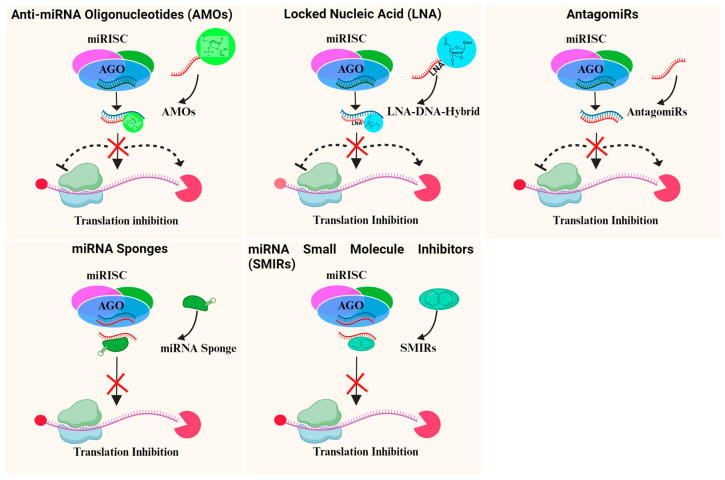
Variety of miRNA inhibition therapies in breast cancer discussed in this review.

The decision to use miRNA-based therapies is because miRNA expression in tumor cells is altered and the tumor phenotype can be altered with miRNA expression modulation. miRNA inhibition therapy consists of the following agents: antisense anti-miRNA oligonucleotides (AMOs), LNA, antagomiRs, miRNA sponges, and miRNA small-molecule inhibitors (SMIRs) [155]. The fundamentals of these approaches are that endogenous miRNAs are isolated in an unrecognizable configuration, resulting in the inactivation and removal of mature miRNAs from the RISC [151,152]. Figure 2 shows the various miRNA inhibition therapies discussed here.

#### 7.1.1. Anti-miRNA Oligonucleotides (AMOs)

AMOs are single stranded chemically modified anti-sense oligonucleotides with a length of 17 to 22 nt and complement the miRNA target of interest. The AMOs target and bind specifically to miRNAs, preventing them from interacting with the mRNAs they are targeting. Inhibiting the function of miRNAs can modulate gene expression and alleviate dysregulated pathways associated with diseases. A significant method for uncovering miRNA biology has been the antisense inhibition of miRNA action. This antisense oligonucleotide was designed to bind to and inhibit the interaction of that miRNA with its unique mRNA targets and the complementary mature miRNA, thus enabling normal translation. Unmodified AMOs, by comparison, are unable to inhibit *in vitro* miRNA function. Okumura et al. stated that modified AMOs targeting miRNA-21 (CL-miR21) interstrand cross-linked duplexes (CLDs) suppressed breast cancer cell proliferation with greater efficiency compared to other types of AMOs. Furthermore, the miRNA-21-controlled expression of tumor-suppressor genes was probably upregulated [156]. In the regulation of miRNAs, CLD-modified AMOs are substantially successful and could be a promising strategy for treating breast cancer. Clinical trials showed some progress for experimental AMO-based drugs directly targeting miRNA-122 in chronic hepatitis C patients (Santaris Pharma-developed Miravirsen and Regulus^®^-developed RG-101) and might be worth considering for therapy of breast cancer [157].

#### 7.1.2. Locked Nucleic Acid (LNA)

LNA is a modified nucleotide with a methylene bridge connecting the 2′-oxygen and 4′-carbon of the ribose sugar ring. As a result, the oligonucleotide becomes more stable and binds better to complementary RNA sequences. LNA-modified AMOs specifically target and inhibit miRNAs. LNA is an example of a modified AMO that binds to miRNA, can modulate miRNA as antisense-based gene silencing, and has recently emerged as a possible miRNA-targeting therapeutic alternative [158]. These are very similar to RNAs in that the methylene bridge in the ribose ring binds the 2′-oxygen atom and the 4′-carbon atom (2′-O 4′-C methylene). This bridge forms a bi-cyclic structure that locks the conformation of the ribose and is integral to its complementary RNA sequences due to the high stability and low toxicity of the biological systems and the affinity of the LNA. In the regulation of RNAs, LNA oligonucleotides are stable, have greater aqueous solubility, and may not induce an immune response, indicating their utility in gene therapy for antisense-based gene silencing [28,158,159]. An *in vivo* study demonstrated that LNA-modified antisense oligonucleotides were capable of silencing upregulated miRNA-205-5p in breast cancer, leading to a significant reduction in tumor growth and metastatic spread in mouse models, thus suggesting the possible future use of this approach in therapy [160].

#### 7.1.3. AntagomiRs

AntagomiRs, also known as anti-miRs, are another class of synthetic oligonucleotides that inhibit the activity of miRNAs, like AMOs. Chemically modified antagomiRs complement the targeted miRNAs with cholesterol-conjugated single-stranded 23-nt RNA molecules. Their backbone consists of single-stranded oligoribonucleotides with 2′-O-methyl (2′-O-Me) and partially modified phosphorothioate linkers [161]. The modifications were made to improve the RNA’s stability and protect it from degradation. The strategy of antagomiRs seems promising for suppressing miRNAs in therapeutic strategies.

The use of antisense miRNAs (antagomiRs) to knockdown miRNAs is one of the most common approaches. Ma et al. demonstrated that systemic treatment of tumor-bearing mice with miRNA-10b antagomiRs suppressed the metastasis of breast cancer, both *in vitro* and *in vivo*. This achieved significant reduction in the levels of miRNA-10b and increased levels of a functionally important miRNA-10b target, *Hoxd10* [25]. In another study, the effect of miRNA-10b antagomiR in a 4T1 mouse model of mammary tumor metastasis was analyzed. AntagomiR-10b systemic delivery had a potent and highly specific metastasis-suppressing effect on these malignant breast cancer cells without affecting their ability to develop as primary tumors; in fact, antagomiR-10b prevented the spread of cancer cells from the primary tumor but did not influence the late stages of the metastatic phase after tumor cells had already disseminated [92]. AntagomiR-21 can affect breast cancer cells by inducing apoptosis and reducing cell proliferation [29]. 

#### 7.1.4. miRNA Sponges

A miRNA sponge is an artificial RNA molecule which binds to miRNAs and prevents them from interacting with their target mRNAs. “miRNA sponges” or “miRNA decoys” offer many synthetic miRNA binding sites which are compete with natural miRNA targets for miRNA binding [162]. The vectors of expression constitute the source of the miRNA sponge transcription, thus reducing the effects of miRNA and increasing the expression of the native targets of miRNA [162]. Because these compounds strongly inhibit target miRNAs and have a high specificity for miRNA sponges, they have been used more frequently in miRNA loss-of-function studies [163]. In one study, a sponge plasmid against miRNA-10b was transiently transfected into MDA-MB-231 and MCF-7, high and low metastatic cell lines. This resulted in the miRNA-10b sponge effectively inhibiting the growth and proliferation of these cell lines [164]. Furthermore, miRNA-21 sponges have been effectively applied to the MDA-MB-231 and MCF-7 breast cancer cell lines [165], while miRNA-9 sponges have been applied to the 4T1 metastatic breast cancer cell line, resulting in a nearly 50% reduction in metastatic activity [92]. Clinically, miRNA sponges and decoys have been developed for more stable suppression and targeted delivery of miRNAs.

#### 7.1.5. miRNA Small-Molecule Inhibitors (SMIRs)

A small-molecule inhibitor of miRNA is a compound designed to modulate the activity of miRNAs. This differs from traditional oligonucleotide-based approaches, which include AMOS and miRNA sponges [166,167]. Due to the decreased time of development, acceptance, and cost, inhibition therapy using SMIRs is an encouraging one. Instead of inhibiting target recognition from miRNA-21, the small molecule’s mode of action is primarily through the transcriptional regulation of miRNA-21. The small molecule enoxacin, an antibacterial fluoroquinolone, has been documented to bind to the miRNA biosynthesis protein TRBP and to increase tumor-suppressor miRNA output [168]. Enoxacin was also shown to inhibit MDA-MB-231 and MCF-7 cell growth [168]. Linifanib, a multi-tyrosine kinase inhibitor, could substantially inhibit miRNA-10b and reverse its oncogenic function in both *in vitro* and *in vivo* breast and liver cancers [169]. A novel strategy for restoring dysregulated miRNAs in cancer is thus defined using small-molecule modulators of miRNAs [153]. Overall, targeting miRNAs for cancer care with SMIRs is an evidence-based approach with high potential and ability for success.

### 7.2. miRNA Replacement Therapy

**Figure 3:**
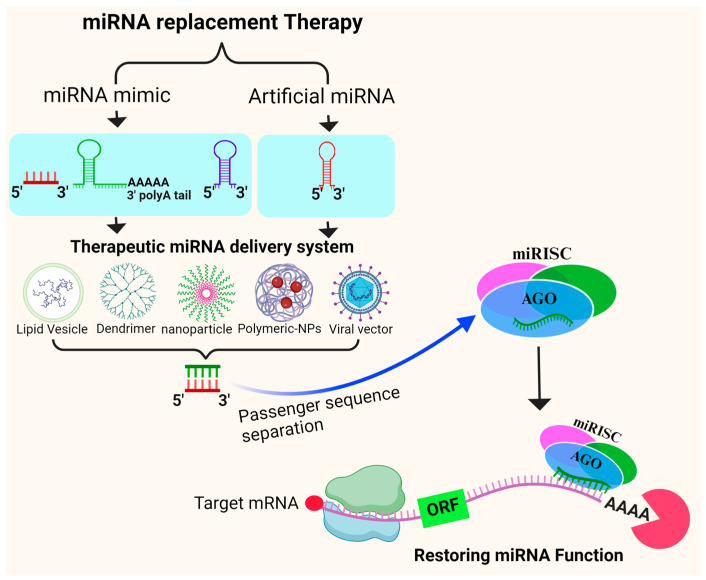
Potential approaches to miRNA replacement therapy for breast cancer using a variety of miRNA delivery systems.

Synthetic miRNA mimics are introduced into cells to restore or enhance the function of a specific miRNA that is deficient or downregulated in breast cancer. By mimicking the function of endogenous miRNAs, this therapeutic approach regulates gene expression and cellular pathways. Synthetic miRNA mimics must be delivered efficiently for target cells for miRNA replacement therapy to be effective. There are a variety of delivery systems that can be used to ensure effective uptake by target tissues, including viral vectors, lipid nanoparticles, and other nanocarriers [150,151,152,170]. A synthetic miRNA mimic is incorporated into the RISC and acts similarly to endogenous miRNAs once inside a cell. Once they bind to target mRNAs, they inhibit the translation or induce the degradation of mRNA, thus regulating gene expression (Figure 3). As a potential treatment strategy for breast cancer, miRNA replacement therapy is being actively explored.

The effects of these therapies may include the suppression of cancer cell growth, modulation of immune responses, and restoration of tissue homeostasis. Tumor cell proliferation can be inhibited, or apoptosis can be induced using miRNA restoration therapy by restoring exogenous tumor-suppressor miRNAs that are downregulated in tumor cells [153]. Different studies have shown the efficacy of *in vitro* and *in vivo* miRNA restoration therapies. For example, Liang et al. introduced miRNA replacement in radioresistant breast cancer cells. For the first time, they showed that miRNA-302a’s enforced expression effectively sensitizes radioresistant tumor cells to irradiation by directly downregulating both *AKT1* and *RAD52* expression [171]. These results suggested that decreased miRNA-302 expression confers radioresistance, and the miRNA-302 baseline expression restoration sensitizes breast cancer cells to radiotherapy.

When miRNA mimics were used to force the expression of miRNA-365 and miRNA-22, breast cancer cell proliferation was inhibited and sensitivity to paclitaxel and fluorouracil was boosted, respectively. By targeting GALNT4, miRNA-365 works on overcoming chemoresistance [172]. In comparison, Jiang et al. found that in breast cancer cells and tissues, miRNA-148a was downregulated, and its overexpression mimics the reduced migration and invasion of breast cancer cells [58]. Also, in breast cancer cells, miRNA-33b expression was downregulated, and it had a negative correlation with the lymph node metastatic status of breast cancer patients. Ectopic overexpression of miRNA-33b inhibited lung metastasis *in vivo* and compromised stem cell characteristics, migration, and invasion *in vitro* in highly metastatic breast cancer cells [52]. MRX34 was involved in the first phase I clinical trial for miRNA replacement therapy and was intended to restore miRNA-34 expression in patients with different solid tumors including TNBC [173]. The use of miRNA-mimetic agents in patients is a promising new way of treating clinical breast cancer.

## 8. Conclusions and Future Directions

To conclude, being able to understand how differentially regulated miRNAs influence breast cancer progression provides valuable insight into their potential diagnostic and therapeutic applications. Unraveling the intricate web of miRNA functions offers valuable insights into their utility as both diagnostic tools and therapeutic targets for breast cancer. By identifying stage- and subtype-specific miRNA signatures, early-detection strategies can be improved and personalized therapeutic interventions can be implemented. Moreover, it is important to determine which miRNA or group of miRNAs is most dysregulated in breast cancer, specifically, at different stages of the disease. Such signatures could serve as robust biomarkers, facilitating more accurate and timely diagnoses, which is crucial for improving patient outcomes. This will assist in identifying and prioritizing the most promising treatment targets, with an emphasis on developing early-detection and -treatment strategies for breast cancer. Better understanding of miRNA-guided networks is essential for improving breast cancer diagnoses and treatment. Ongoing research in this field holds the promise of translating miRNA-based discoveries into clinically relevant applications, fostering advancements in breast cancer management.

## Figures and Tables

**Table 1:** List of miRNAs and the function of their targets in breast cancer.

MicroRNA	Target	Function	References
Oncogenic miRNAs in breast cancer			
miRNA-10b	*HOXD10*	Promotes cell migration, invasion, and metastasis	[25]
miRNA-17/92 cluster	*COL4A3*, *LAMA3*, *TIMP2/3*, *ADORA1*	Promotes lymph node metastasis, enhanced cell proliferation, colony formation, migration, and invasion in Triple Negative Breast Cancer (TNBC)	[26,27]
miRNA-21	*PDCD4*, *PTEN, TPM1*, *TIMP3*	Promotes invasion, metastasis, and migration	[28,29,30,31]
miRNA-24	*Nanog*, *Oct-3/4*, *BimL*, *F1H1*, *HIF-1*, *Snail*, *VEGFA*	Hypoxia-inducible miRNA	[32]
miRNA-122	Pyruvate kinase and citrate synthase	Promotes metastasis by reprogrammed glucose metabolism	[33]
miRNA-135b	*LATS2*, *CDK2*, *p-YAP*	Promotes cell proliferation and S-G2/M cell cycle progression	[34]
miRNA-155	*SOCS1*, *TP53INP1*, *FOXO3*	Promotes cell growth, proliferation, and survival	[35,36,37]
miRNA-181a	*Bim*	Promotes epithelial-to-mesenchymal transition (EMT), migration, and invasion	[38]
miRNA-191-5p	*SOX4*, caspase-3, caspase-7, *p53*	Promotes apoptosis resistance and doxorubicin resistance	[39]
miRNA-200b	*Ezrin/Radixin*/*Moesin* (*ERM*)	Promotes metastasis and invasion	[40]
miRNA-206	*NK-1*	Promotes breast cancer cell invasion, migration, proliferation, and colony formation *in vitro*	[41]
miRNA-210	*Pax-5*	Modulating EMT and hypoxia	[42]
miRNA-331	*HER2*, *HOTAIR*, *E2F1*, *DOHH*	Promotes metastasis and invasion by elevation in plasma of metastatic breast cancer patients	[43]
miRNA-373	*CD44*	Promotes cell migration, invasion, and metastasis	[44]
miRNA-455-3p	*EI24*	Promotes proliferation, invasion, and migration	[45]
miRNA-498	*BRCA1*	Promotes TNBC cell proliferation	[46]
miRNA-520c	*CD44*	Promotes cell migration, invasion, and metastasis	[44]
Tumor-suppressor miRNAs in breast cancer			
miRNA-17-92	*Mekk2*, *cyclin D1*	Promotes NK cell antitumoral activity and reduces metastasis, regulates G1 to S phase transition	[47,48]
miRNA-7	*SETDB1*	Inhibits cell invasion and metastasis, decreases the BCSC population, and partially reverses EMT	[49]
let-7d	*Cyclin D1*	Induces stem cells radiation sensitization	[50]
miRNA-30	*Ubc9*, *ITGB3*	Inhibits self-renewal of breast tumor-initiating cells (BT-ICs), trigger apoptosis	[51]
miRNA-33b	*HMGA2*, *SALL4*, *Twist1*	Regulates cell stemness and metastasis	[52]
miRNA-34a	*IMP3*	Regulates TNBC stem cell property	[53]
miRNA-125b	*ERBB2, EPO*, *EPOR*, *ENPEP*, *CK2-α*, *CCNJ*, *MEGF9*	Inhibits cell proliferation and differentiation, migration and invasion	[54,55]
miRNA-137	*FSTL1*	Suppresses TNBC stemness	[56]
miRNA-143	*ERK5*, *MAP3K7*, *Cyclin D1*	Anti-proliferative	[57]
miRNA-148a	*WNT1*, *MMP13*	Inhibits cell proliferation, migration and invasion	[58,59]
miRNA-200a	*TFAM*	Regulates breast cancer cell growth and mtDNA copy number	[60]
miRNA-203	*ΔNp63α*	Forfeiture of self-renewing capacity associated with epithelial stem cells, suppresses proliferation and colony formation	[61]
miRNA-205	*HMGB3*, *Notch-2*	Suppresses proliferation and invasion and inhibits EMT and stem cell properties	[62,63]
miRNA-206	*Cyclin D2*, *Cx43*	Reduces migration, invasion, and metastasis	[64,65]
miRNA-223	*HAX-1*	Re-sensitizes TNBC stem cells to tumor necrosis factor-related apoptosis	[66]
miRNA-483-3p	*Cyclin E1*, *p-NPAT*, *NPAT*, *CDK2*	Anti-proliferative and G1-S cell cycle arrest	[67]
miRNA-497	*Cyclin E1*	Anti-proliferative and reduces migration	[68]
miRNA-519d-3p	*LIMK1*	Suppresses growth and motility	[69]

**Table 2:** Characteristics of miRNAs in subtypes of breast cancer.

Subtype	miRNA Signature	Findings	Analysis Type	References
Luminal-A	miRNA-99a/let-7c/ miRNA-125b	High expression in Luminal A compared to Luminal B tissue	Breast cancer tissues-cluster analysis of small RNAseq	[107]
Luminal-A	miRNA-221	High expression	Plasma samples—analyzed using qRT-PCR	[108]
Luminal-A	miRNA-16, miRNA-21, miRNA-155, miRNA-195	Higher expression than healthy controls	Serum circulating miRNAs analyzed using qRT-PCR	[109]
Luminal-A	miRNA-29a, miRNA-181a and miRNA-652	Reliably differentiate between cancers and controls	Microarray analysis and confirmed using qRT-PCR	[110]
Luminal-A	miRNA-206	Higher expression	Serum samples and analyzed using qRT-PCR	[111]
Luminal-B	miRNA-342	Higher expression	Early-stage breast cancer specimens— microarray and qRT-PCR analysis	[112]
Luminal-B	miRNA-182-5p and miRNA-200b-3p	Significantly higher than in normal breast tissues	Breast cancer tissue: global focus miRNA PCR Panel and analyzed using qRT-PCR	[113]
Luminal-B	miRNA-21, miRNA-221, miRNA-200a and miRNA-196a	Overexpression in Luminal B breast cancer without overexpression of HER2/neu	Tumor tissues of patients with Luminal B breast cancer and analyzed using qRT-PCR	[114]
Luminal-B	miRNA-210	Upregulated in the analyses of all 40 patients’ samples	FFPE blocks: miRNA microarray and confirmed using qRT-PCR	[115]
Luminal-B	miRNA-222	Higher expression in Luminal-B/(HER2^+^) subtypes than in Luminal A and TNBC subtypes	Breast cancer tissues and analyzed using qRT-PCR	[116]
HER2-enriched	miRNA-125b	Significantly increased expression in breast cancer tissues compared with that in the non-cancerous tissues	FFPE breast cancer tissues, luciferase activity and qRT-PCR	[117]
HER2- enriched	miRNA-375 and miRNA-122	High levels of circulating miRNA-375 and low levels of miRNA-122 were associated with HER2 status	Serum specimens: Solexa deep sequencing and confirmed using qRT-PCR	[118]
HER2- enriched	miRNA-548ar-5p, miRNA-584-3p, miRNA-615-3p, and miRNA-1283	Significantly differential expression in the HER2-enriched subtype	Serum samples: Multiplexed gene expression analysis	[119]
HER2- enriched	let-7f, let-7g, miRNA-107, miRNA-10b, miRNA-126, miRNA-154 and miRNA-195	Inversely correlated with HER2 overexpression	Primary breast cancer biopsies: microarray and confirmed using qRT-PCR	[120]
HER2- enriched	miRNA-520d, miRNA-376b	Highly expressed and accurately predicted HER2 status in early-stage breast tumors	Early-stage breast cancer specimens- microarray and qPCR analysis	[112]
TNBC or basal-like	miRNA-210	Significantly higher expression compared to ER^+^/HER2^−^ breast cancers	Breast cancer tissue and analyzed using qRT-PCR	[121]
TNBC or basal-like	miRNA-214	Higher expression	FFPE tissues and confirmed using qRT-PCR	[122]
TNBC or basal-like	miRNA-493	High expression	Tissue microarrays	[123]
TNBC or basal-like	miRNA-135b	High expression	TaqMan low-density array	[124]
TNBC or basal-like	miRNA-20a, miRNA-221	Higher expression compared to Luminal A and Luminal B/HER2^−^ breast cancer subtypes	Biopsies of tumor tissue; and confirmed using qRT-PCR	[125]
TNBC or basal-like	miRNA-17 family	Overexpressed in high grade and TNBC associated with aggressive behavior	FFPE- tissue: miRCURY LNA microarray and confirmed using qRT-PCR	[126]
TNBC or basal-like	miRNA-17-92	Elevated in TNBC but reduced in ER-positive breast cancer and associated with poor outcome. The miRNA-17–92 expression enhanced cell growth and invasion of TNBC cells	Breast cancer cell lines: high-throughput mRNA sequencing and confirmed using qRT-PCR	[27]
